# Impact of Parenchymal Hemorrhage on Internal Carotid Artery Wall Shear Stress in Post-Traumatic Vasospasm: A Dynamic Helical CTA Study

**DOI:** 10.21203/rs.3.rs-10092671/v1

**Published:** 2026-06-28

**Authors:** Alexey O. Trofimov, Gregory N Vlasov, Kseniia A Trofimova, Ilya V Koshcheev, Dmitry A Martynov, Anastasia V Kivenko, Vladislav V Romanychev, Edwin M Nemoto, Denis E Bragin

**Affiliations:** Privolzhsky Research Medical University: Privolzskij issledovatel’skij medicinskij universitet Ministerstva zdravoohranenia Rossijskoj Federacii; Regional Hospital; Privolzhsky Research Medical University: Privolzskij issledovatel’skij medicinskij universitet Ministerstva zdravoohranenia Rossijskoj Federacii; Privolzhsky Research Medical University: Privolzskij issledovatel’skij medicinskij universitet Ministerstva zdravoohranenia Rossijskoj Federacii; Alekseev Nizny Novgorod State Technical University: Nizegorodskij gosudarstvennyj tehniceskij universitet imeni R E Alekseeva; Privolzhsky Research Medical University: Privolzskij issledovatel’skij medicinskij universitet Ministerstva zdravoohranenia Rossijskoj Federacii; Regional Hospital; New Mexico State University; Lovelace Biomedical

**Keywords:** Wall Shear Stress1, Traumatic Brain Injury2, Cerebral Vasospasm3, Computed Tomography Angiography Source Image4, Neurohemodynamics5

## Abstract

**Background:**

Post-traumatic cerebral vasospasm (CVS) significantly worsens outcomes in moderate-to-severe traumatic brain injury (msTBI). While wall shear stress (WSS) modulates endothelial function, its state in post-traumatic CVS remains poorly understood. We aimed to assess supraclinoid internal carotid artery (ICA) WSS in msTBI patients with and without non-surgical parenchymal hemorrhage.

**Methods:**

This retrospective cohort study (2013–2024) included 85 adults with msTBI and angiographic CVS. Patients were divided into Group 1 (Marshall II-III, no hematoma; n=43) and Group 2 (Marshall IV, non-surgical parenchymal hemorrhage; n=42). Dynamic helical CT angiography (DHCTA) and Doppler ultrasound were performed 2–5 days post-injury. WSS in the ICA C7 segment was calculated using a Poiseuille-based formula incorporating mean CBFV and vessel radius.

**Results:**

ICA WSS significantly exceeded the reference range (0.82 ± 0.08 Pa) in both groups (p<0.001). In Group 2, WSS was significantly higher than in Group 1 (p<0.0001). Specifically, Group 2 ipsilateral WSS (64.4 ± 9.8 Pa) was significantly higher than both the contralateral side (49.3 ± 8.3 Pa; p=0.021) and the Group 1 mean (33.2 ± 7.5 Pa; p<0.0001). No significant age-WSS correlation was observed (p=0.72). Group 2 exhibited a trend toward worse clinical outcomes (GOS), though only moderate disability (GOS 4) showed a significant difference (p<0.05).

**Conclusions:**

Post-traumatic CVS in msTBI is associated with markedly elevated ICA WSS. The presence of parenchymal hemorrhage further amplifies this hemodynamic stress, potentially contributing to non-physiological microcirculatory remodeling. These findings suggest WSS could serve as a biomarker for secondary brain injury risk, necessitating individualized surgical and medical management.

## Introduction

Traumatic brain injury (TBI) remains a pervasive cause of neurological disability worldwide, with recent data indicating an 18.2% lifetime prevalence of consciousness-altering TBI among adults in the United States. In absolute numbers, this means at least 64 million Americans have suffered a TBI in their lifetime [1]. The Centers for Disease Control and Prevention (CDC) data show even higher estimates: approximately 3,042,000 new cases of TBI were reported in the United States in 2024, and the overall prevalence of concussion or TBI among adults ranges from 19% to 29% [2]. A gender-population analysis of TBI showed a threefold predominance of men over women (73.9% versus 26.1%), and an analysis of the causes of TBI indicated a prevalence of road traffic accidents, which accounted for 69.5% of traumatic brain injuries [3].

Despite significant progress in research since the early 21st century, recent meta-analyses indicate that our understanding of TBI pathogenesis remains far from complete [4]. Although modern studies (such as the CBI-M) have expanded our approaches by introducing the concept of “TBI modifiers,” such as biomarkers, age, and other comorbidities that influence injury progression [5], it is certain that cerebral microcirculation disorders play a leading role in the development of secondary strokes. The development of circulatory disorders in the vasa vasorum system, endothelial dysfunction, and vascular wall edema increase vascular stiffness and decrease arterial compliance, reduce critical closure pressure, increase resistance, and prolong the time constant [6]. Increased peroxidation, driven by the release of iron ions during subarachnoid hemorrhage, leads to the formation of peroxides and nitrous oxide, which damage the endothelium, intima, and elastic membranes [7]. Studies of transmural water and oxygen transport in the vascular wall demonstrate that it depends on intraluminal blood flow [8], while wall shear stress (WSS) in pulsating vessels modulates hydraulic conductivity and oxygen permeability. Previously, WSS dynamics were mainly studied in pathologies of the coronary and carotid basins [9]. However, WSS disturbances in moderate-to-severe TBI (msTBI), especially in cases of cerebrovascular vasospasm (CVS) without intracranial hematomas, were associated with arterial wall changes due to subarachnoid hemorrhage and remain poorly understood. The aim was to study the changes of cerebral WSS in posttraumatic vasospasm in moderate-to-severe TBI patients with and without non-surgical parenchymal hemorrhage.

## Materials and Methods

### Study Design and Population

The retrospective cohort non-randomized single-center study analyzed a previously collected database (2013–2025). The study protocol was approved by the Institutional Local Ethical Committee, and informed consent was waived due to the retrospective nature of the study.

The inclusion criteria were as follows: (1) moderate-to-severe TBI (Glasgow Coma Score (GCS) <11 points); (2) mild polytrauma (Injury Severity Score (ISS) <9 points); (3) parenchymal brain lesion (Marshall score II-IV); (4) multiphase dynamic helical computed tomography angiography (DHCTA) and Doppler ultrasound (2–5 days after trauma); (5) “angiographic” CVS on available computed tomography angiography source image (CTASI); (6) unilateral CVS in anterior cerebral circulation (anterior or middle cerebral artery, or inner carotid artery).

The exclusion criteria were as follows: (1) blast and/or penetrate TBI; (2) age younger than 18 years and older than 65 years; (3) serum creatinine values >120 mg/L; (4) ISS > 9 points; (5) Glasgow Coma Score (GCS) < 5 and > 12 points (to focus on severe but potentially survivable injuries, excluding profound coma or mild TBI cases); (5) Marshall scores I (absence of brain damage) or V-VI (neurosurgical brain damage); (6) any performed neurosurgical operations or non-evacuated epidural or subdural lesion; (7) bilateral and/or CVS in posterior cerebral circulation.

We examined 85 patients with moderate-to-severe TBI and CVS due to subarachnoid hemorrhage (SAH) who were treated at the Nizhny Novgorod Trauma Center Level I in 2013–2024. The mean age was 34.5 ± 11.2 years (range: 19–65 years), with 42 women and 43 men.

Patients matching the inclusion criteria were divided into two groups. Group 1 included patients with Marshall Score II-III. Group 2 included patients with a Marshall Score of IV. Group 1 (n=43, men – 22, women – 21, mean age was 37.1 ± 9.3 years) had no intracranial hematoma, while Group 2 (n=42, men – 21, women – 21, mean age was 31.2 ± 14.3 years) included patients with parenchymal hemorrhages.

### Dynamic Helical Computed Tomography Angiography

DHCTA was performed 2–5 days after TBI (mean 3.1 ± 0.5 days) using a CT scanner (Aquilion Prime SP, TSX 303B, Canon Medical Systems Europe B.V., the Netherlands. Scan parameters were: pitch 0.625mm × 80; 120 kVp, 100 mAs; collimation 80 × 0.5 mm, effective dose = 3.3 mSv; rotation time 0.5 s; and scan range 188.0mm, respectively. A bolus of contrast agent (Ultravist 370, Schering AG, Germany) was injected using an automatic injector (Dual Shot Alpha7, Nemoto, USA) at a rate of 5 mL/s and a volume of 50 mL into a peripheral vein of the right arm (primarily - the right antecubital vein). Scan data were transferred to PACS (KIR, RF), Vitrea FX (Vitrea, Vital Images, USA) workstations, and MATLAB 2025b (The MathWorks Inc., Natick, USA). Arterial and venous markers were automatically placed and manually verified (arterial marker – anterior cerebral artery, venous marker – posterior third of the upper sagittal sinus). Cluster analysis was then used to monitor the profiles on the contrast agent concentration-time plot.

**Figure 1.**
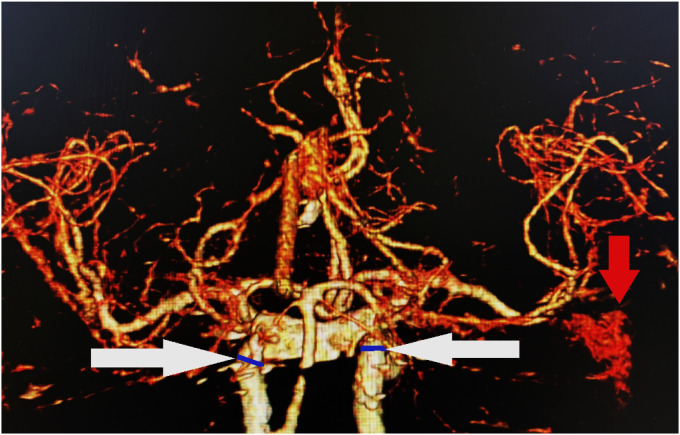
3D reconstruction of the multidetector computed tomography angiography (straight view). The white arrows point to the supraclinoid segment (C7) of both internal carotid arteries, where their radii were measured (marked with a blue line). The red arrow points to the parenchymal component of the intracerebral hemorrhage.

The multiplane computed tomography angiography analysis was performed to visualize the supraclinoid segments of both ICAs and to assess their lumens) (Figure 1).

The DHCTA data analysis revealed a regional narrowing of the internal carotid artery (ICA) diameter exceeding 30% compared to adjacent sections, leading to the diagnosis of an “angiographic” CVS. The severity of CVS was classified: mild grade – <30% narrowing of luminal diameter; moderate – >50% reduction of the lumen; severe – >70% of the luminal narrowing [10]. All CT data were reviewed for CVS by two experienced neuroradiologists, who were blinded to clinical conditions.

### Multimodal Neuromonitoring

Immediately after DHCTA, cerebral blood flow velocities (CBFVica) in both internal cerebral arteries were recorded using 2-MHz Doppler ultrasound probes (Sonomed 300M, Spectromed, RF). The criteria for establishing a “dopplerographic” CVS (mean CBFV in the middle cerebral artery>120 cm/sec and Lindegaard index>3) were not used due to the need for a planimetric assessment of the area of both ICAs,

Arterial blood pressure was measured non-invasively (Life Pulse 110, Huntleigh Healthcare Ltd, UK). The mean ICA diameters were measured on DHCTA volumes in the С7 segments of both ICA as close as possible to the cavernous sinus. The average WSS (in dynes per square centimeter) was determined based on Poiseuille’s law. For a constant laminar flow in a vessel, the WSS was calculated using the modified formula [11]:

WSS=4×CBFVica×a/R,

where a is the viscosity and R is the radius of the tube (in cm). The blood density and viscosity were estimated at 1.0 g/mL and 3.45 cPa, respectively, and the WSS reference range was set to 0.82 ± 0.08 Pa [12,13].

### Statistical Analysis

Statistical analysis was done using Statistica 12 software (TIBCO Software Inc., Palo Alto, USA). Data were evaluated for normality using the Shapiro-Wilk criterion. Comparisons employed Student’s T-criterion as appropriate. Data are presented as mean ±standard deviations (mean±SD). The significance level was preset at p < 0.05

## Results

### Clinical Parameters and Outcomes

We found no mean age difference between Group 1 and Group 2 (37.1 ± 9.3 years versus 31.2 ± 14.3 years, respectively; p > 0.05). The wakefulness levels during CT scanning, according to GCS, were similar (11.3 ± 1.4 versus 10.1 ± 0.9, respectively; p=0.43). The Injury Severity Score (ISS) severity at admission was also comparable (6 ± 2 versus 6 ± 3, respectively, p=0.86).

Clinical outcomes according to the Glasgow Outcome Scale (GOS) are shown in Table 1. Group 2 exhibited a trend toward worse outcomes (e.g., GOS 1 (Death) 33.3% versus 27.9%, p>0.05; GOS 2 (Vegetative state) 7% versus 9.5%, p>0.05; GOS 3 (Higher Severe Disability) 35.7% versus 25.6%, p>0.05; GOS 5 (Good Recovery) 20.9% versus 16.7%, p>0.05), though differences were not significant. However, the differences between GOS 4 outcome (Moderate Disability) were significant (18.6% versus 4.8%, p<0.05).

### Hemodynamic and ICA Planimetric Parameters

Hemodynamic parameters and ICA planimetric assessments are summarized in Table 2.

We did not find any significant differences in MAP between the Group 1 and Group 2 (79.1±11.4 mmHg versus 84.8±12.5 mmHg, respectively, p>0.05).

The CBFV_ICA_ differences between the Group 1 and Group 2 were significant (78±12 cm/sec, 101±11 cm/sec, 112±23 cm/sec, respectively, p<0.001). Meanwhile, the differences in CBFV_ICA_ between the ipsi-and contralateral sides in Group 2 were not significant (p>0.05).

The differences in ICA diameters were statistically significant only between the Group 1 and the ipsilateral side of parenchymal hemorrhage in Group 2 (0.31±0.08 cm, 0.24±0.03 cm, respectively, p=0.042). Any other differences in ICA diameters were not significant (p>0.05).

The wakefulness levels according to GCS were similar (11.3 ± 1.4 versus 10.1 ± 0.9, respectively, p=0.43). The Injury Severity Score (ISS) severity at admission was also comparable (6 ± 2 versus 6 ± 3, respectively, p=0.86).

Wall shear stress (WSS) in the ICA C7 segment significantly exceeded the reference range (0.82 ± 0.08 Pa) in both groups (p<0.001).

**Figure 2.**
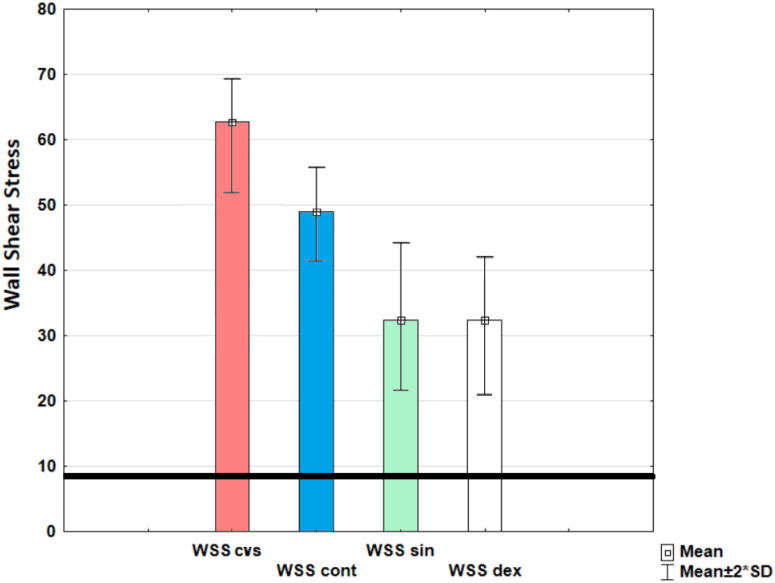
Comparison of WSS (in Pa) in Group 1 and Group 2. WSS sin and WSS dex – WSS values in Group 1 on the left (green box) and right side(white box), respectively. WSS cvs and WSS CONT – WSS values in Group 2 on the CVS side (red box) and the contralateral side, respectively. Error bars represent standard deviations. Reference WSS is shown as a solid black line.

In Group 2, WSS was significantly higher on the ipsilateral CVS side (64.4 ± 9.8 Pa) and contralateral side (49.3 ± 8.3 Pa) compared to Group 1 (33.2 ± 7.5 Pa) and the normal range (p<0.0001). Ipsilateral WSS in Group 2 was also significantly higher than contralateral WSS (49.3 ± 8.3 Pa) (p=0.021) (Figure 2). No significant age-WSS correlation was observed (p=0.72).

## Discussion

Our study demonstrated a close association between post-traumatic cerebral vasospasm and a significant increase in wall shear stress in large cerebral arteries. The highest WSS values were recorded ipsilateral to the vasospasm focus. These data suggest that elevated WSS is not simply a passive consequence of vessel narrowing but also an active participant in the pathological cascade. Understanding the clinical significance of these changes requires comparing them with known molecular and cellular mechanisms previously described in the literature [14].

Much of the fundamental knowledge about the influence of WSS on the vascular wall has been gained from studies of coronary and peripheral arterial circulation. Research in this area has convincingly demonstrated that spatial and temporal changes in WSS modulate atherogenesis and endothelial function. Although studies of WSS have primarily focused on atherosclerotic lesions of the heart and coronary arteries, they have shown that elevated WSS is significantly associated with abnormal expression of interleukin-1β (IL-1β) and tumor necrosis factor (TNF-α) [15]. These cytokines induce the expression of adhesion proteins (VCAM-1, ICAM-1) and chemokines in the vascular endothelium, promoting the adhesion of leukocytes from the bloodstream to the vascular wall, a key event in the development of inflammation [16].

The effects of WSS have been shown to be dose-dependent and not always linear. Importantly, high WSS values reduce the ability of TNF-α or IL-1β to activate the endothelium and suppress the proinflammatory response [17]. In the context of the coronary artery, a high laminar WSS is considered a protective factor, maintaining the endothelium in a quiescent, anti-inflammatory state. In our study, despite high WSS values, we observed vascular spasm—a condition associated with endothelial dysfunction. This discrepancy can be explained by several factors. First, under conditions of acute injury and ischemia, the endothelium may lose its normal mechanosensitivity. Second, in cerebral vessels, the threshold values for “pathological” and “physiological” WSS may differ from those in the coronary arteries. Third, against the backdrop of a powerful proinflammatory environment caused by TBI itself and blood breakdown products, the protective mechanisms of a high WSS may be suppressed [18].

### WSS and Regulation of Vascular Tone

The central mechanism linking WSS to vascular wall health is the endothelium’s regulation of vasoactive factor production. As shown by Gijsen et al. [19], WSS is involved in regulating endothelial and vascular function through the release of nitric oxide (NO) and endothelin-1 (ET-1). Normally, an increase in WSS is a potent stimulus for endothelial NO synthase (eNOS), leading to NO production and vasodilation (the mechanism underlying flow-dependent dilation) [20]. Simultaneously, the production of the potent vasoconstrictor ET-1 is suppressed.

However, in pathology, this balance is disrupted. Given their opposing effects on vascular tone, microcirculation remodeling, as well as angiogenesis and collateral formation, adequate tissue perfusion is maintained and secondary injury is prevented [21]. Under conditions of post-traumatic vasospasm and ischemia, the endothelium may lose the ability to adequately produce NO (due to eNOS dysfunction or NO inactivation by free radicals such as oxyhemoglobin). At the same time, ET-1 production, stimulated by inflammatory cytokines (TNF-α, IL-1β), thrombin, and the mechanical stress itself, can persist or even increase. In this situation, high WSS loses its vasodilatory properties and can paradoxically contribute to the maintenance or worsening of vasoconstriction and stimulate smooth muscle cell proliferation, thereby promoting vessel remodeling [22].

Our data on the prolonged (up to 5 days) maintenance of high WSS values specifically on the injured side fits well with this model. Surgical decompression and vasospasm lead to a dramatic change in local hemodynamics. In a constricted vessel with a reduced diameter, blood flow velocity (as determined by TCD) increases sharply, which causes peak WSS values. This hemodynamic “stress” is superimposed on the already damaged, inflammation-sensitized endothelium, creating a “vicious cycle” of endothelial dysfunction [23].

Elevated WSS persisting for several days after injury may serve as a marker of high risk for persistent vasospasm and delayed cerebral ischemia. Hemodynamic stress in the area of vasospasm may explain the predisposition to infarction in arterial territories adjacent to the injury [24].

Although studies on coronary arteries demonstrate the protective role of high laminar WSS, this mechanism appears to be impaired in cerebral arteries in TBI complicated by vasospasm [25]. The developed methodology may form the basis for the creation of new prognostic models to identify patients at the highest risk of hemodynamically mediated vascular wall injury and, consequently, those requiring more aggressive prevention of secondary ischemia. Further research is needed to understand the molecular correlates of these hemodynamic changes.

### Limitations

This study has several limitations. First, the relatively small sample size and retrospective design. Second, building computed tomography angiography–based computational fluid dynamics models requires high segmentation accuracy and is time-consuming, which currently limits the method’s applicability in routine clinical practice [26]. Third, we did not conduct direct correlations between WSS values and plasma or CSF levels of endothelial dysfunction biomarkers (NO, ET-1, cytokines). Including such laboratory correlates in future studies would allow confirmation of the molecular mechanisms postulated based on the literature [27].

## Conclusion

The anterior circulation CVS in msTBI significantly increases WSS in the ipsilateral side of the main cerebral artery (ICA). Furthermore, intracerebral hemorrhage (ICH) associated with vasospasm, even small volume, that do not require surgical removal, leads to an even greater increase in ICA, which (1) indicates its role in the development of a non-physiological type of cerebral microcirculation remodeling that provokes secondary brain damage, and (2) raises the question of the need for an individualized approach to the surgical treatment of ICH associated with CVS.

## Figures and Tables

**Table 1:** Clinical outcomes (GOS) of msTBI patients

GOS Category	Description	Group 1	Group 2
GOS 1	Death	12 (27.9%)	14 (33.3%)
GOS 2	Vegetative state	3 (7%)	4 (9.5%)
GOS 3	Severe disability	11 (25.6%)	15 (35.7%)
GOS 4	Moderate disability	8 (18.6%)	2 (4.8%)
GOS 5	Good recovery	9 (20.9%)	7 (16.7%)
Total		43 (100%)	42 (100%)

**Table 2:** Comparison of the analyzed parameters

Groups	MAP (mm Hg)	CBFVica (cm/sec)	ICA diameter (cm)	WSS (Pa)
Group 1	79.1±11.4	78±12	0.31±0.08	33.2 ± 7.5
Group 2 (ipsilateral side)	84.8±12.5	101±11	0.28±0.07	49.3 ± 8.3
Group 2 (contralateral side)	84.8±12.5	112±23	0.24±0.03	64.4 ± 9.8
P (1–2)	0.723	0.023*	0.067	<0.001*
P (1–3)	0.723	0.003*	0.042*	<0.0001*
P (2–3)	-	0.892	0.823	0.021*

* Difference is statistically significant, MAP – mean arterial pressure, CBFVica – cerebral blood flow velocity in ICA, ICA diameter - diameter of internal carotid artery, WSS – wall shear stress

## Data Availability

The raw data supporting the conclusions of this article will be made available by the authors without undue reservation.
